# A hotspots analysis-relation discovery representation model for revealing diabetes mellitus and obesity

**DOI:** 10.1186/s12918-018-0640-4

**Published:** 2018-12-14

**Authors:** Guannan He, Yanchun Liang, Yan Chen, William Yang, Jun S. Liu, Mary Qu Yang, Renchu Guan

**Affiliations:** 1 Key Laboratory of Symbolic Computation and Knowledge Engineering of Ministry of Education, College of Computer Science and Technology, Jilin University, Changchun, 130012 China; 2 Zhuhai Laboratory of Key Laboratory of Symbolic Computation and Knowledge Engineering of Ministry of Education, Zhuhai College of Jilin University, Zhuhai, 519041 China; 3 Department of Endocrinology, The Second Hospital of Jilin University, Changchun, 130000 China; 4 Department of Computer Science, Carnegie Mellon University, Pittsburgh, PA 15213 USA; 5 Department of Statistics, Harvard University, Cambridge, MA 02138 USA; 6 MidSouth Bioinformatics Center and Joint Bioinformatics Ph.D, Program of University of Arkansas at Little Rock and Univ. of Arkansas Medical Sciences, 2801 S. Univ. Ave, Little Rock, 72204 USA; 7 University of Arkansas at Little Rock, Little Rock, AR 72204 USA

**Keywords:** Representative latent Dirichlet allocation, Diabetes mellitus, Obesity

## Abstract

**Background:**

Nowadays, because of the huge economic burden on society causing by obesity and diabetes, they turn into the most serious public health challenges in the world. To reveal the close and complex relationships between diabetes, obesity and other diseases, search the effective treatment for them, a novel model named as representative latent Dirichlet allocation (RLDA) topic model is presented.

**Results:**

RLDA was applied to a corpus of more than 337,000 literatures of diabetes and obesity which were published from 2007 to 2016. To unveil those meaningful relationships between diabetes mellitus, obesity and other diseases, we performed an explicit analysis on the output of our model with a series of visualization tools. Then, with the clinical reports which were not used in the training data to show the credibility of our discoveries, we find that a sufficient number of these records are matched directly. Our results illustrate that in the last 10 years, for obesity accompanying diseases, scientists and researchers mainly focus on 17 of them, such as asthma, gastric disease, heart disease and so on; for the study of diabetes mellitus, it features a more broad scope of 26 diseases, such as Alzheimer’s disease, heart disease and so forth; for both of them, there are 15 accompanying diseases, listed as following: adrenal disease, anxiety, cardiovascular disease, depression, heart disease, hepatitis, hypertension, hypothalamic disease, respiratory disease, myocardial infarction, OSAS, liver disease, lung disease, schizophrenia, tuberculosis. In addition, tumor necrosis factor, tumor, adolescent obesity or diabetes, inflammation, hypertension and cell are going be the hot topics related to diabetes mellitus and obesity in the next few years.

**Conclusions:**

With the help of RLDA, the hotspots analysis-relation discovery results on diabetes and obesity were achieved. We extracted the significant relationships between them and other diseases such as Alzheimer’s disease, heart disease and tumor. It is believed that the new proposed representation learning algorithm can help biomedical researchers better focus their attention and optimize their research direction.

**Electronic supplementary material:**

The online version of this article (10.1186/s12918-018-0640-4) contains supplementary material, which is available to authorized users.

## Background

In today’s era of obesity, contributing to the increasing risk of many chronic diseases, such as diabetes, cancer, and cardiovascular diseases, it is quickly becoming one of the greatest public health challenges [1, 2]. From 1980 to 2013, it provides a 41% increase in the population of overweight [3]. Of all the obesity co-morbidities, diabetes account for the strongest correlation [4]. Meanwhile, both obesity and diabetes impose large economic burdens on society [5]. Therefore, researches on diabetes and obesity are becoming more and more important to human health and biomedical research. They have become the worldwide prevalent and harmful metabolic diseases, which bring the pain to patients and stimulate the researchers and clinicians constantly. In 2007, with a genome-wide association (GWA) study conducted by Frayling, the rs9939609 polymorphism, located in the first intron of the FTO gene, was proved strongly associated with type 2 diabetes mellitus and obesity [6]. This discovery explains the reason of the co-occurring nature of diabetes mellitus and obesity. Moreover, due to their genetic characteristics, diabetes and obesity occur along with other diseases, such as cardiovascular diseases and metabolic syndrome, is also found in clinical medicine [7]. Although some papers have discussed about which diseases are associated with diabetes and obesity [8–10], there is no quantitative analysis of the relationships between diabetes, obesity, and other diseases. Moreover, to the best of our knowledge, there is also a lack of artificial intelligence tool to pick out the hotspots for the diabetes and obesity research of each year.

With the fast development of biotechnology and genome research [11, 12], a huge amount of biomedical literatures and data are published in digital libraries such as National Center for Biotechnology Information and The Cancer Genome Atlas. Especially for diabetes and obesity study, hundreds of thousands papers were published in the last 10 years. For example, in 2016, 49,804 papers or reports about diabetes and obesity were published in PubMed. However, facing the increasing massive biomedical literature, it will cost plenty of time and human efforts to read and understand them. It is a challenge for clinician or biological researchers to quickly obtain the cutting-edge information and research problems from such massive literature with effective techniques. To solve this problem efficiently, machine-learning technologies provides us effective ways [13]. For example, conditional random fields (CRFs) is proven to be effective in named entity recognition [14], latent Dirichlet allocation (LDA) has been applied in sentiment analysis [15], and Native Bayes methods excellently performed on large amount of text classification [16]. However, there is no representation learning approach is designed for diabetes mellitus and obesity topics modeling.

In this paper, to discover meaningful relationships from the large collections of literature, more than 300,000 abstracts and titles of diabetes mellitus and obesity literatures in the past 10 years (2007~ 2016) from PubMed have been collected. These data contain the most valuable information for hotspots revealing. Therefore, a novel model named as representative latent Dirichlet allocation (RLDA) is designed to discover the important relationships between diabetes mellitus, obesity and other diseases and search significant topics for them. Furthermore, by analyzing the trend of research based on the past decade, the hotspots in the near future can also be identified.

## Results

Firstly, we introduce the experiment dataset and show the prepossessing steps such as data collection and name entity selection. Then, based on experiment results, we performed an explicit analysis to find the relationships between diabetes mellitus, obesity and other diseases. Furthermore, we achieved proofs from the clinical reports, which were exclusive in RLDA training process. In addition, the inference results of diabetes mellitus and obesity research hotspots expected in the near future are shown.

**Fig. 1:**
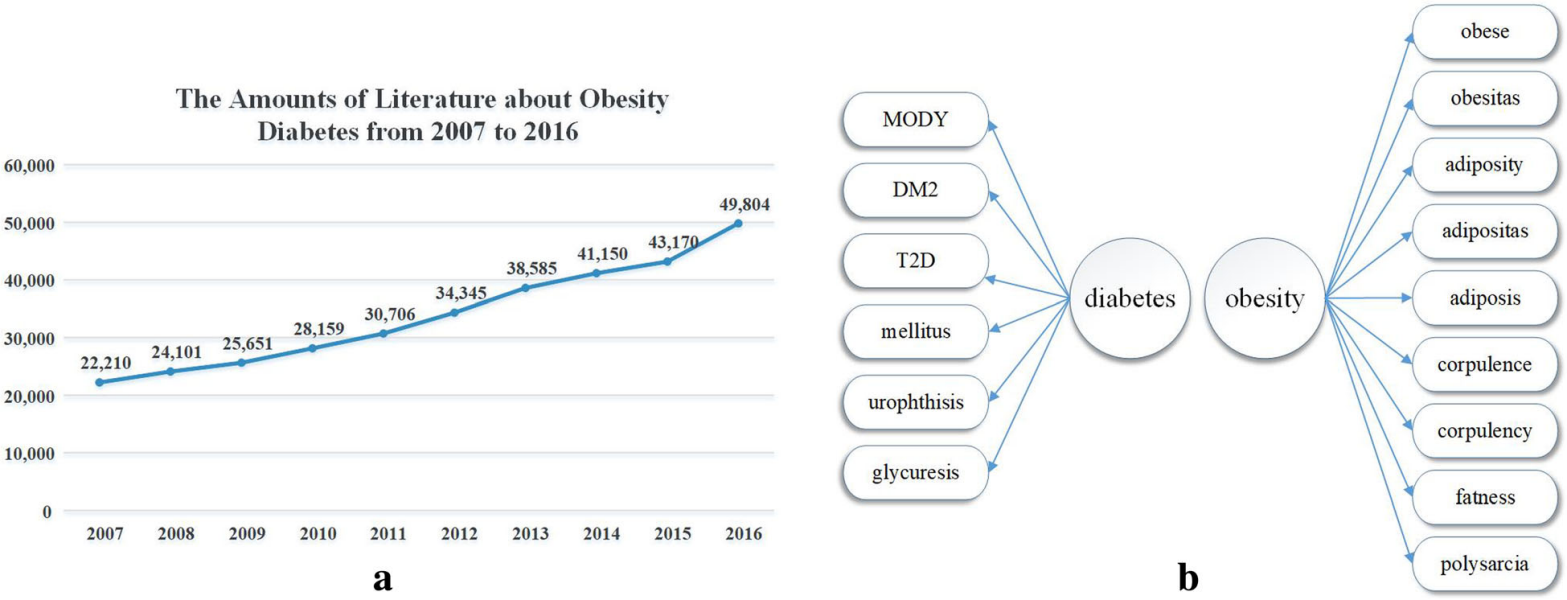
**a** shows the amounts of literature about obesity and diabetes from 2007 to 2016. The amounts increase year by year, and the total amount of ten years is 337,881. **b** shows the synonyms of diabetes and obesity. Diabetes has 6 synonyms, such as MODY, DM2, T2D, mellitus, urophthisis and glycuresis. Obesity has 9 synonyms, such as obese, obesitas, adiposity, adipositas, adiposis, corpulence, corpulency, fatness and polysarcia

**Fig. 2:**
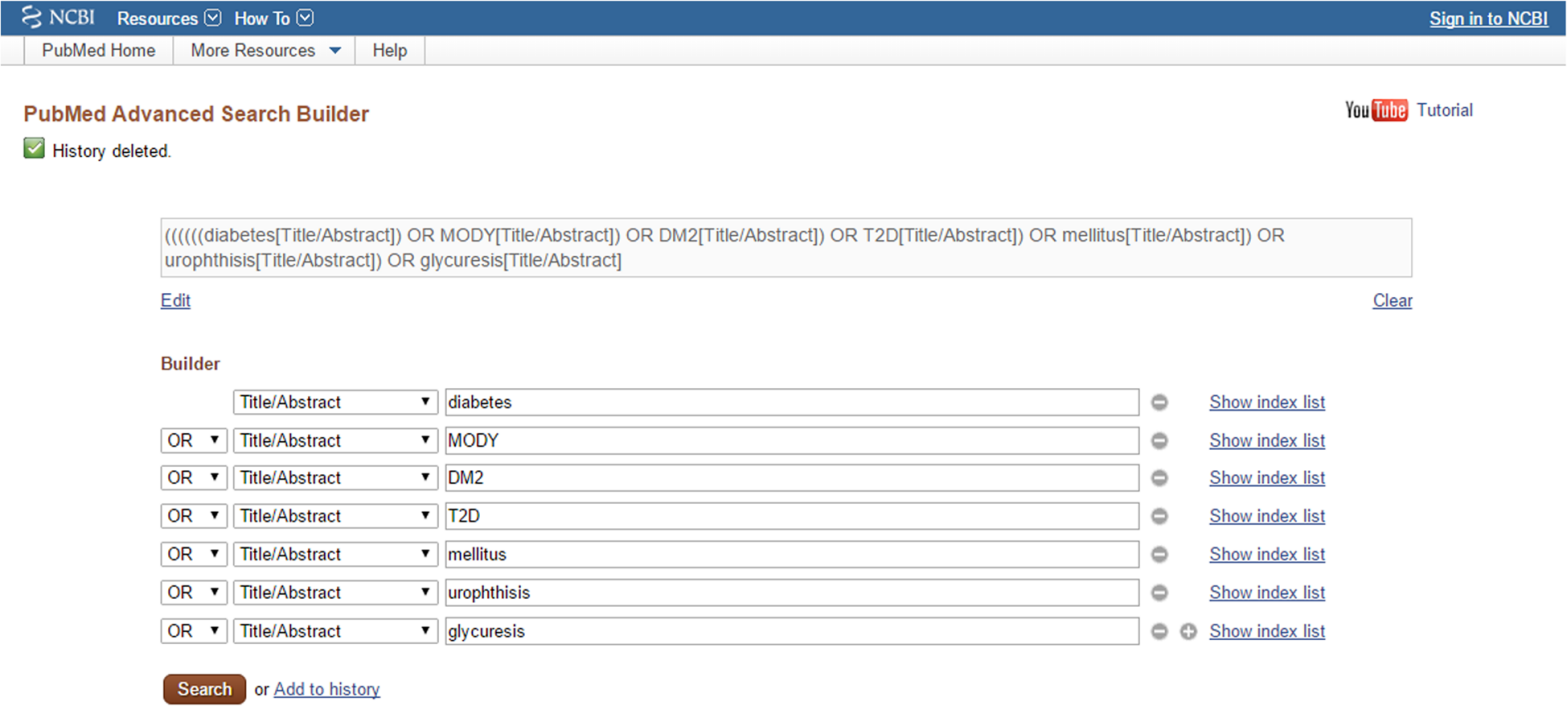
A query builder of PubMed for diabetes is shown as an example. We input all the synonyms of diabetes into the query builder at once. The condition logic is “OR” and the search field is “Title/Abstract”

**Fig. 3:**
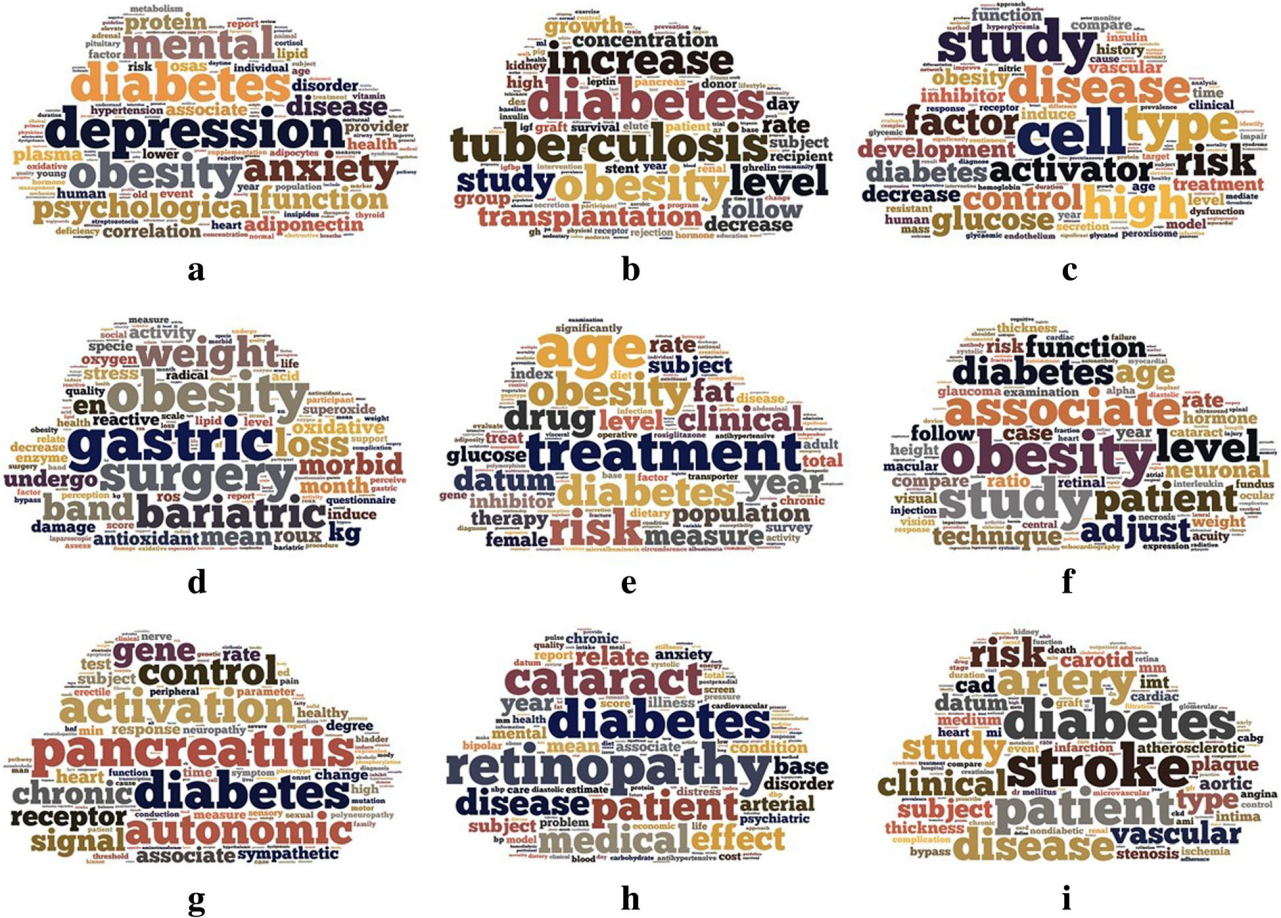
The word cloud results of 2008. The representative central topic words can be separately summarized as “depression”, “tuberculosis”, “cell”, “gastric”, “treatment”, “obesity”, “pancreatitis”, “retinopathy” and “stroke”. From these figures, we can reach the result that pancreatitis, retinopathy, cataract and stroke usually appear with diabetes in the research papers of diabetes mellitus; gastric disease often appear with obesity; tuberculosis, psychological or mental disease like depression and anxiety appear with both diabetes and obesity frequently

Titles and abstracts of literature about diabetes or obesity published in the past 10 years (2007~ 2016) were downloaded from PubMed. The entity names “diabetes” and “obesity” as well as their synonyms are shown in Fig. 1b. We input all the synonyms of diabetes into the search form of PubMed to build a query for research literature about diabetes, as shown in Fig. 2. The same method was used for obesity. The amounts of literature for each year are shown in Fig. 1a. After text segmentation, lemmatization, and stop words removing we input the pre-processed data into our proposed representative latent Dirichlet allocation topic model (RLDA). To get a deeper understanding, we need an effective tool, which can visualize the RLDA results. Word cloud is employed to display different size of words, the higher the word weight is, the bigger the word is. The bigger one word is, the more important role it plays. Taking the result of 2008 as an example, RLDA model produces nine clusters, and the central topic words are summarized as “depression”, “tuberculosis”, “cell”, “gastric”, “treatment”, “obesity”, “pancreatitis”, “retinopathy”, and “stroke” as shown in Fig. 3. In the word cloud diagram of our results, every word represents the core of the topics’ cluster, and each cluster indicates the related research about diabetes mellitus or obesity. In Fig. 3a, depression is the central word that can represent the whole clu ster of diabetes and obesity topics. The other obvious words such as *mental*, *anxiety*, and *psychological* also exactly associate with depression. Therefore, we reached the conclusion that there is a non-ignorable relationship between psychological or mental diseases such as depression and anxiety and obesity and diabetes mellitus. Hereinto, depression topic is a hotspot on diabetes in 2008. However, not all the word cloud diagrams are help to our analysis. We cannot obtain any relationship between diabetes mellitus, obesity and other diseases from some figures in 2008, such as Fig. 3c, e, f.

We made the analysis on other clusters of 2008 in the same way, and more discoveries were achieved. The new findings unveiled that pancreatitis, retinopathy, cataract, and stroke are closely asscociated with diabetes. Gastric disease is related with obesity. Moreover, hypertension, myocardial infarction and tuberculosis are closely asscociated with both diabetes mellitus and obesity. More word cloud results of other years are shown in Additional file 1. Figure S1.

**Fig. 4:**
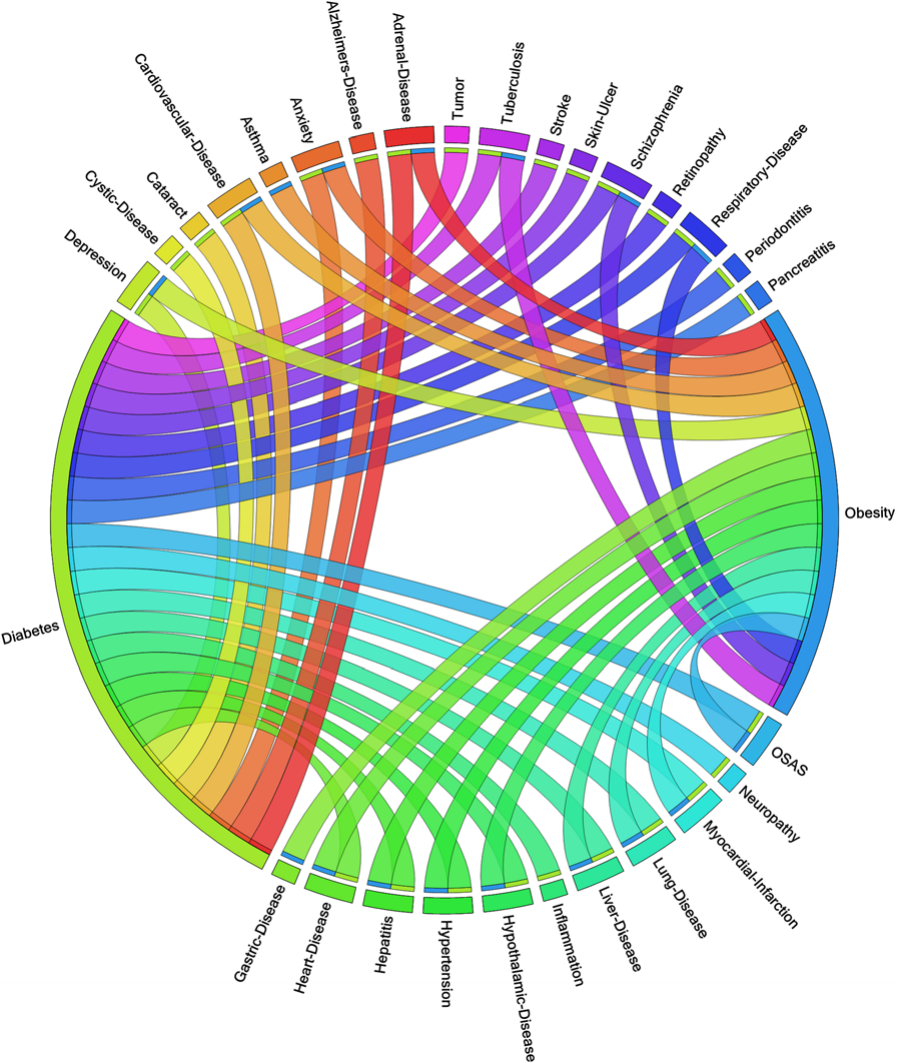
The chord diagram of relationships between diabetes, obesity and other diseases is shown in this figure. Each segment represents a disease and each ribbon represents that there is a relationship between the two diseases which are linked by the ribbon. We can clearly see that 26 diseases which have relationships with diabetes, 17 with obesity and 15 with both (Adapted with permission from [44])

For the last decade data, we found more interesting associations between diabetes mellitus, obesity and some other diseases. In Fig. 4, to show the experiment results vividly, we draw a direct chord diagram based on the 10 years’ discoveries. In Fig. 4, the two longer segments are diabetes mellitus and obesity; the 24 shorter segments indicate 24 related diseases; and the ribbons define the relationship between the two diseases. Each short piece is linked to at least one long segment when there is a relationship between them, e.g. the segment labeled “Tumor” is linked to “Diabetes” to show tumor is associated with diabetes. Several short segments such as hypertension and heart disease include two parts, which connect both “Diabetes” and “Obesity”. It means that these segments have relationships with both diabetes mellitus and obesity. In the last 10 years, obesity study is mainly focused on 17 accompanying diseases, adrenal disease, anxiety, asthma, cardiovascular disease, depression, gastric disease, heart disease, hepatitis, hypertension, hypothalamic disease, liver disease, lung disease, tuberculosis, myocardial-infarction, OSAS (obstructive sleep apnea syndrome), respiratory disease and schizophrenia. For diabetes, a large scope including 26 diseases from adrenal disease, Alzheimer’s disease, anxiety, cardiovascular disease, cataract, cystic disease, depression, heart disease, hepatitis, hypertension, hypothalamic disease, inflammation, liver disease, neuropathy, OSAS, pancreatitis, periodontitis, respiratory disease, retinopathy, schizophrenia, skin ulcer, stroke, tuberculosis, lung disease, myocardial infarction, and tumor. Furthermore, there are 15 diseases having relationships with both of diabetes and obesity, i.e. adrenal disease, anxiety, cardiovascular disease, depression, heart disease, hepatitis, hypertension, hypothalamic disease, myocardial infarction, liver disease, lung disease, OSAS, respiratory disease, schizophrenia and tuberculosis.

## Results proof

As Ananiadou warned, although using widely applied algorithms, in our case latent Dirichlet allocation, Word2vec and affinity propagation, and the large-scale text collections, how to estimate the correctness of the results is still a critical problem [17]. For our experiments results, we demand that they can be proved with strong evidences. Therefore, we employ the authoritative clinical reports about diabetes and obesity in recent years, such as Standards of Medical Care in Diabetes - 2016 [18] and The State of Obesity: 2016 [19]. They were excluded in our dataset. The solid research reports will prove our discovered relationships are correct and significant for clinical researches and RLDA is effective for discovery searching from massive literatures. With the activation of these results, this model can also benefit those researchers who continuously devote themselves to study diabetes mellitus and obesity.

For diseases significant associated with diabetes mellitus, take depression, myocardial infarction, retinopathy, cataract, stroke, hypertension, hepatitis and heart disease as examples, the details of the diseases, quotes, and clinical reports are shown in Table.1. Other relationships and proofs are shown in Additional file 1.Table S2. For obesity study, take asthma, heart disease, hypertension and liver disease as examples, their proofs for our discoveries (i.e.significant relationships) are shown in Table.2 and Additional file 1.Table S3.

**Table 1:** Clinical Report Proofs on the Discoveries about Diabetes and Other Diseases (Reproduced with permission from [45])

Diseases	Quotes	Clinical Report
**Depression**	**Depression** affects 20–25% of people with diabetes.	Standards of Medical Care in Diabetes - 2016 [18]
**Myocardial infarction**	Individuals with both diabetes and major depressive disorder have a twofold increased risk for new onset **myocardial infarction** compared with either disease state alone.	
**Retinopathy**	Diabetic **retinopathy** is a highly specific vascular complication of both type 1 and type 2 diabetes.	
**Cataract**	Glaucoma, **cataracts**, and other disorders of the eye occur earlier and more frequently in people with diabetes.	
**Stroke**	Older individuals with diabetes have higher rates of premature death, functional disability, and coexisting illnesses, such as **hypertension**, coronary heart disease, and **stroke**, than those without diabetes.	
**Hypertension**		
**Hepatitis**	Compared with the general population, people with type 1 or type 2 diabetes have higher rates of **hepatitis** B.	
**Heart disease**	Almost 50% of patients with type 2 diabetes will develop **heart failure**.	

The "bold" words are the match information in clinical report

**Table 2:** Clinical Report Proofs on the Discoveries about Obesity and Other Diseases (Adapted with permission from [45])

Diseases	Quotes	Clinical Report
Depression	Obese adults are more ikely to have **depression**, **anxiety** and other mental health.	The State of Obesity 2016 [19]
Anxiety		
Asthma	Being overweight or obese can put children at a higher risk for health problems such as **heart disease**, **hypertension**, type 2 diabetes, stroke, cancer, **asthma** and osteoarthritis — during childhood and as they age.	
Heart disease		
Hypertension		
Liver disease	Up to 25% of adults have nonalcoholic fatty **liver disease** (NFLD), which can lead to liver damage (cirrhosis) or the need for transplants.	

The "bold" words are the match information in clinical report

## Methods

**Fig. 5:**
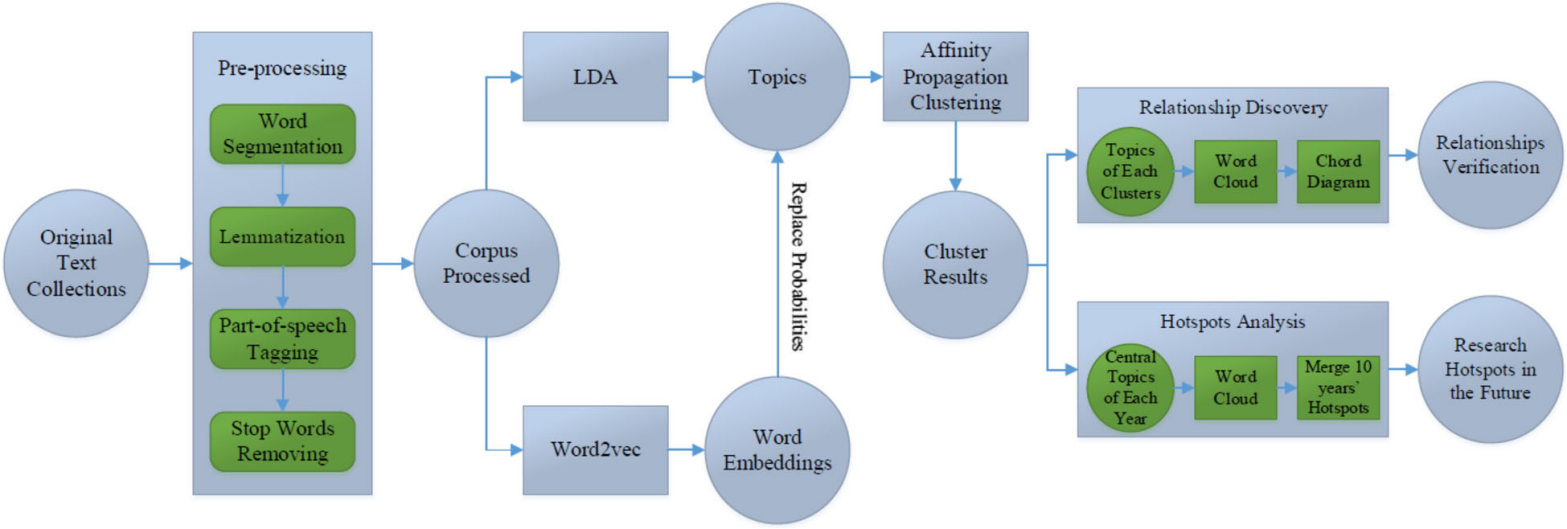
Representation latent Dirichlet allocation (RLDA) model

To reveal relationships and extract research hotspots, a novel model named as representation latent Dirichlet allocation (RLDA) based on LDA topic model, word2vec and affinity propagation clustering. Its flowchart is shown as Fig. 5.

### Pre-processing

Because the raw biomedical literatures contain noisy information (such as stop words) that has little contribution to the result and even be harmful, before revealing relationships, we applied word segmentation, lemmatization, part-of-speech tagging and stop words removing to pre-process the biomedical texts, and finally got clean corpus.

Word segmentation can separate the text into several tokens by punctuations. After the segmentation, lemmatization is to transform various forms of one word into prototype. For example, “men” is the plural form of “man”, lemmatization can change the plural of a noun into its singular form. Another example, “walked” and “walking” should be restored to their prototype “walk”. Then, part-of-speech tagging was applied to assign every word a tag and the tags are shown in Table 3. As nouns and adjectives are often considered overweigh other words in topical semantics [20], we extracted nouns and adjectives as our corpora. However, there are still a lot of meaningless words in raw data such as “is”, “and”, “the”, “at” and so on which have no influence on the semantic of the sentences. Finally, stop words removing is applied which is also a common step in pre-processing [21, 22]. It removed the useless words from text collection, including coordinating conjunctions, cardinal numbers, prepositions, pronouns and so on except nouns and adjectives.

**Table 3:** Part-of-speech Tags in Pre-processing

Tag	Description	Examples	Tag	Description	Examples
CC	Coordinating conjunction	and, or	PRP	Personal pronoun	I, you, he
CD	Cardinal number	one, two	PRP$	Possessive pronoun	your, one’s
DT	Determiner	a, the	RB	Adverb	quickly, never
EX	Existential ‘there’	there	RP	Particle	up, off
FW	Foreign word	mea culpa	SYM	Symbol	+, %, &
IN	Preposition/sub-conj	of, in, by	TO	“to”	to
JJ	Adjective	good, long	UH	Interjection	ah, oops
LS	List item marker	1, 2, One	VB	Verb, base form	look, eat
MD	Modal	can, should	WDT	Wh-determiner	which, that
NN	Noun	apple, book	WP	Wh-pronoun	what, who
NNP	Proper noun	IBM	WP$	Possessive wh-	whose
PDT	Predeterminer	all, both	WRB	Wh-adverb	how, where
POS	Possessive ending	‘s			

### LDA topic model

Recently, probabilistic topic models have been extensively developed. It turns out that these models have a very excellent performance on text mining. The classical topic model, latent Dirichlet allocation which was proposed by David M. Blei in 2003 is an unsupervised topic model based on probability and statistics [23]. LDA is an extremely effective topic model which can be applied to large-scale and complex text data to mine meaningful latent topic information [24, 25]. From the moment that LDA was proposed, it was continuously developed and has been widely applied to document summarization [26], sentiment analysis [27], thematic structure revealing [28] and so on.

LDA is a Bayesian statistical model and involves three structures, words, topics and documents. It supposes that each word of a document is selected from a topic with a certain probability and this topic is also chosen from this document with a certain probability [29]. A topic is a distribution of terms over the vocabulary, which allows each document to be represented as a distribution over topics. It can be expressed by the Eq. (1). Let *d* be a document, *w* indicate a word, *t* be a topic.

1 $$ P\left(w\left|d\right.\right)=P\left(w\left|t\right.\right)\times P\left(t\left|d\right.\right) $$

LDA assembles a document collection *D* = {*d*_*m*_}_*m*∈{1…*M*}_ with a fixed vocabulary *W*. Let φ_*k*_ indicate the distribution of probabilities that all words belong to topic *t*_*k*_, and *θ*_*m*_ indicate the distribution of probabilities that all topics belong to document *d*_*m*_. Therefore, the distribution of topic *k* over vocabulary is defined as Φ = {φ_*k*_}, *k*∈{1,…,*K*}, and the distribution of the *m*th document over all *K* topics is defined as Θ = {*θ*_*m*_}, *m*∈{1,…,*M*}. For document *m*, the distribution of document over topics *θ*_*m*_ and the distribution of topics over vocabulary Φ are sampled from prior *α* and *β*, respectively. The topic assignment *z* for each word is generated from *θ*_*m*_; the accurate words *w* are got according to their respective topic assignment *z* and the distribution of topics over Φ. The joint distribution of this model can be simply expressed by Eq. (2) which describes its generative process. *N*_*m*_ is the length of document *m*, and *z*_*m,n*_ is the generating topic in document *m*.

2 $$ p\left({w}_m,{z}_m,{\theta}_m,\Phi \left|\alpha, \beta \right.\right)=\prod \limits_{n=1}^{N_m}p\left(\Phi \left|\beta \right.\right)p\left({\theta}_m\left|\alpha \right.\right)p\left({z}_{m,n}\left|{\theta}_m\right.\right)p\left({w}_{m,n}\left|\Phi, {z}_{m,n}\right.\right) $$

To solve the priori probability problem, we use Gibbs sampling, a random sampling method, to estimate LDA model and infer the result [30].

In this work, we applied LDA model to each year’s data. With several adjustments, we set the topic number *t* = 100, hyper-parameters *α* = 0.05 which commonly equals 5/*t*, *β* = 0.01 which the same as [20], and the iteration *i* = 500. The output matrix of LDA contains 100 rows and 20 columns. Each row represents a topic, each column is a word and its probability in this topic. In each topic, we took the top 20 words which are sorted by their probabilities in descending order. The probability represents how much this word belongs to the topic, the same word may have different probabilities in different topics. Thus, we can’t directly use the matrix of probability to measure the similarities between each pair of topics.

### Word2vec

Word2vec is a group of versatile distributed representation learning models based on a three-layer neural network, which is first proposed by Mikolov [31]. It can project text data to a k-dimensional vector space and represent words as word embeddings. The closer semantics the corresponding words have, the more similar the two vectors are [32]. Recently, plenty of NLP tasks, such as knowledge graph completion and text mining have introduced word2vec model [33–35].

By exploiting word2vec, the word embeddings and semantic relationships among words are learned from large amount of text corpus. This method is derived from neural probabilistic language model [36]. It contains two neural architectures: Skip-gram and continuous bag of words (CBOW) models [32]. They employ two different training techniques: hierarchical softmax and negative sampling [37]. Both of these two models have three layers: input, projection and output layer. The CBOW architecture predicts the current word based on the context, and the Skip-gram predicts surrounding words by the given current word. The optimizing process is done using stochastic gradient descent (SGD) method. Recently, word2vec has significantly outperformed traditional language models in many research areas, such as sentiment analysis [38], text classification [39] and semantic analysis [40]. Furthermore, Word2vec is an unsupervised model which doesn’t need labels, and given enough text corpus, it can produce meaningful representations of words. In our experiments, we used Skip-gram model and training method.

We train word2vec model on the data of each year respectively. Word2vec model mapped all the words to word embeddings in the same semantic space. Afterwards, we replaced every word’s probability in the LDA result with its corresponding word embedding, thus each topic became a matrix, and the result of LDA model became a three-dimensional tensor.

### Affinity propagation clustering algorithm

Affinity propagation (AP) algorithm is a widely-used clustering model based on “message passing” among data points. Different from K-means or K-medoids, AP algorithm does not require the exact number of clusters before clustering. AP finds “exemplars”, which are real samples of the input, as the representatives of clusters [41]. It has been used in image processing [42], gene detecting [43], text mining [44] and so on.

This algorithm supposes a sample set *X* = {*x*_1_, *x*_2_, … *x*_*n*_} without inner structure between sample points. Let *S* be the similarity matrix of samples, for example, *s*(*i*, *j*) indicate the similarity of point *x*_*i*_ and *x*_*j*_. The similarity can be set different metrics according to different applications. In our experiment, the similarity between two topics matrices (*X*_*i*_, *X*_*j*_) is the negative reciprocal of cosine similarity corresponding to Eq.(3). To avoid the case that *cosθ* equals zero, we add a minimal value *x* to it. We calculated the weighted average of the rows the in two matrices for computing the *cosθ to* Eq.(4) and the weights are the probabilities of the words in topics.

3 $$ S=\left\{\begin{array}{c}-\frac{1}{\cos \theta },\cos \theta \ne 0\\ {}-\frac{1}{\cos \theta +x},\cos \theta =0\end{array}\right. $$

4 $$ \cos \theta =\frac{\sum_{k=1}^l\left({x}_{ik}\times {x}_{jk}\right)}{\sqrt{\sum_{k=1}^l{x}_{ik}^2}\times \sqrt{\sum_{k=1}^l{x}_{jk}^2}} $$

AP clustering algorithm defines two matrices, one of which is responsibility matrix *R* (*r*[*i*, *k*]) representing the degree of sample *k* suitable as the cluster center of sample *i*, and another is availability matrix *A* (*a*[*i*, *k*]) representing the degree of sample *i* choosing sample *k* as its cluster center. The matrix *R* will be constantly updated according to Eq.(5), and the matrix *A* according to Eq.(6) and Eq.(7) [41].

5 $$ \mathrm{r}\left(i,k\right)=s\left(i,k\right)-\underset{k\hbox{'}\ne k}{\max}\left\{a\left(i,{k}^{\hbox{'}}\right)+s\left(i,{k}^{\hbox{'}}\right)\right\} $$

6 $$ \mathrm{a}\left(i,k\right)=\min \left(0,r\left(k,k\right)+\sum \limits_{i\hbox{'}\notin \left\{i,k\right\}}\max \left\{0,r\left({i}^{\hbox{'}},k\right)\right\}\right),i\ne k $$

7 $$ \mathrm{a}\left(k,k\right)=\sum \limits_{i\hbox{'}\ne k}\max \left\{0,r\left({i}^{\hbox{'}},k\right)\right\} $$

To avoid numerical oscillations, the algorithm introduces an damping factor *λ* (*λ* ∈ (0,1)) when updating the two matrices corresponding to Eq.(8) and Eq.(9).

8 $$ {r}_t\left(i,k\right)\leftarrow \left(1-\lambda \right){r}_t\left(i,k\right)+\lambda {r}_{t-1}\left(i,k\right) $$

9 $$ {a}_t\left(i,k\right)\leftarrow \left(1-\lambda \right){a}_t\left(i,k\right)+\lambda {a}_{t-1}\left(i,k\right) $$

We applied AP algorithm to each year’s topics to get the “exemplars” as the centers of clusters. Every cluster is our analysis target to discover relationships between diabetes, obesity and other diseases.

## Discussion

The hotspots on diabetes mellitus and obesity research are evolving for each year. However, there are some latent tendencies under them. Detecting the research trend is one of our aims, which is significant for researchers to easily focus and adjust their future research.

### Research Trend detection

**Fig. 6:**
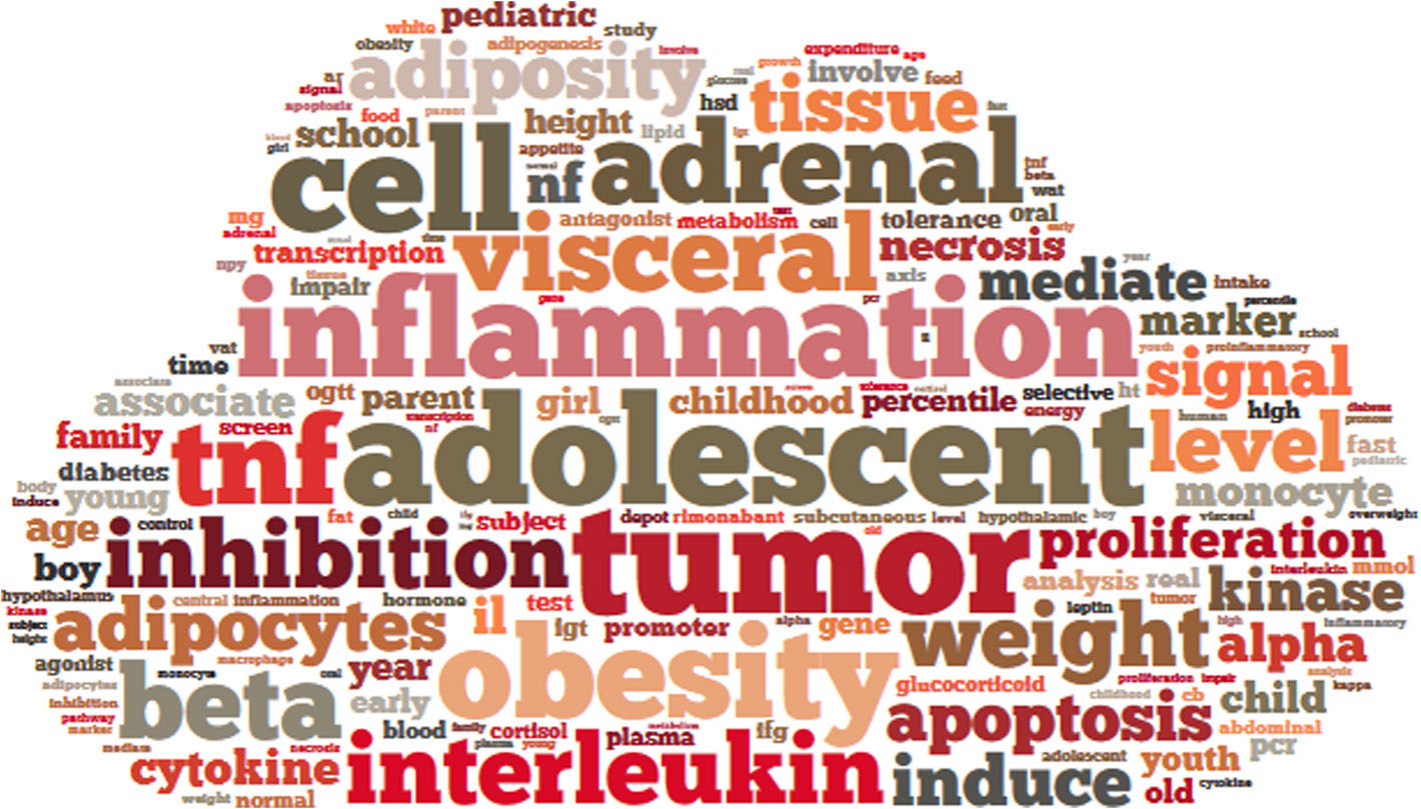
The word cloud figure of research hotspots in 2007 shows that the most conspicuous words, such as “adolescent”, “tumor”, “inflammation”, “tnf”, “cell”, “adrenal”, “interleukin” and “visceral”, are research hotspots about diabetes and obesity in 2007 due to their highest frequencies of appearing in literature

To visualize the words of cluster centers, we applied word cloud charts. To capture the research hotspots for each year, we merge all the central topics of the whole year into a super word cloud. Taking the data of 2007 as an example, the visualization result is shown Fig. 6. From this figure, we can get that with their high frequencies, tumor, adolescent, tnf, inflammation, cell, adrenal, interleukin and visceral are the most conspicuous words. These eight words are considered as the 2007 research hotspots. The other hotspots figures of 2008 ~ 2016 are shown in Additional file 1.Figure S4.

**Fig. 7:**
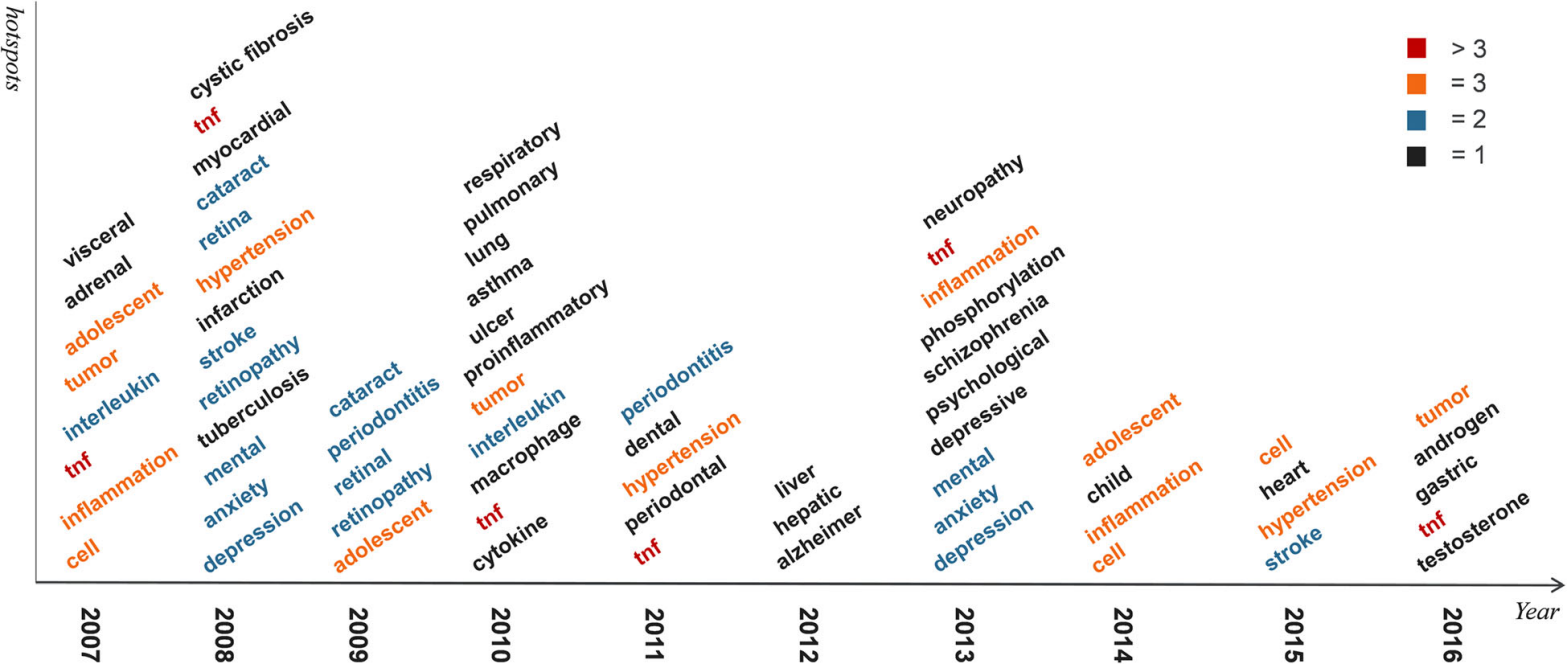
Research hotspots of every year from 2007 to 2016 are summarized in this figure. The words which appear more than thrice are marked by red, those appear thrice are marked by orange, those appear twice are marked by blue, and those appear only once are marked by black. (Adapted with permission from [44])

In Fig. 7, the cluster central topics for all 10 years are shown, which are identified as the research hotspots for each year. The central topical words are ranked by their appearance frequencies to unveil the underlying tendency. The result is shown in Table.4 in which we put the semantically similar words together and several findings can be clearly achieved as follows:

1. “Tnf” has the maximum times of appearance, and is the hotspot almost every year.
2. “Tumor”, “inflammation”, “hypertension”, “adolescent” and “cell” appeared three times in the last 10 years. Moreover, they are hotspots in the latest 3 years (2014~ 2015).
3. The other hotspots appear changeably, and the times of appearances are less than three.

**Table 4:** Hotspots of Diabetes Mellitus and Obesity Research for the Past Decade (Adapted with permission from [45])

Research Hotspots	Appearance Times	Appearance Years
tnf (tumor necrosis factor)	6	2007,2008, 2010,2011, 2013,2016
Tumor	3	2007, 2010, 2016
Inflammation	3	2007, 2013, 2014
Hypertension	3	2008, 2011, 2015
Adolescent	3	2007, 2009, 2014
Cell	3	2007, 2014, 2015
Cataract/retina/retinopathy	2	2008, 2009
Stroke	2	2008, 2015
Mental/anxiety/depression	2	2008, 2013
Periodontitis	2	2009, 2011
Interleukin	2	2007, 2010

Therefore, with their contribution to diabetes mellitus and obesity research for the past decade, we can find that tnf, tumor, adolescent obesity or diabetes, inflammation, hypertension and cell are potentially going to be the hot topics in the very near future.

## Conclusions

To reveal the hotspots of diabetes mellitus and obesity research and find out the significant relationships between these two diseases and others, we proposed a novel model representative latent Dirichlet allocation topic model (RLDA). It is a reasonable combination of several effective models containing LDA, word2vec and AP. Massive bio-medical published literature in the past decade (2007~ 2016) is downloaded from PubMed with key words of these two diseases as well as their synonyms. We applied RLDA to extract the topical words of each cluster and discover the diseases that are closely associated with diabetes and obesity. From the 10 years’ data, we totally discovered 26 diseases are significantly associated with diabetes, 17 with obesity and 15 with both. To prove the discoveries and the effectiveness, we achieved related research proofs from recent years’ clinical reports which are not included in our training data. In addition, we studied the research hotspots of via a visualization method to find the regularity, and give a revelation of the research hotspots on diabetes mellitus and obesity in the very near future. The results show that RLDA using massive text data is significant and helpful to researchers. We are going to apply RLDA to other complex diseases such as cancer.

## Additional file

Additional file 1: Word cloud results of ten years and clinical report proofs on relationships between diabetes, obesity and other diseases. (PDF 10960 kb)

## Abbreviations

AP: Affinity Propagation
CBOW: Continuous bag of words
CRFs: Conditional random fields
GWA: Genome-wide association study
LDA: Latent Dirichlet allocation
OSAS: Obstructive sleep apnea syndrome
RLDA: Representative latent Dirichlet allocation topic model
SGD: Stochastic gradient descent
Tnf: Tumor necrosis factor
