# Supplementary material for: A hotspots analysis-relation discovery representation model for revealing diabetes mellitus and obesity
Source: BMC Syst Biol. 2018 Dec 14;12(Suppl 7):116. doi: 10.1186/s12918-018-0640-4 (PMC6293500; doi:10.1186/s12918-018-0640-4)
Supplement: Supplementary file 1 — Word cloud results of ten years and clinical report proofs on relationships between diabetes, obesity and other diseases. (PDF 10960 kb) [file 12918_2018_640_MOESM1_ESM.pdf]

Supplementary of a hotspots analysis-relation discovery representation model for revealing diabetes mellitus and obesity

Guannan He[1], Yanchun Liang[1,2], Yan Chen[3], William Yang[4], Jun S. Liu[5], Mary Qu Yang[6], Renchu Guan[1, 7,*]

[1] Key Laboratory of Symbolic Computation and Knowledge Engineering of Ministry of Education, College of Computer Science and Technology, Jilin University, Changchun 130012, China

[2] Zhuhai Laboratory of Key Laboratory of Symbolic Computation and Knowledge Engineering of Ministry of Education, Zhuhai College of Jilin University, Zhuhai 519041, China

[3] Department of Endocrinology, The Second Hospital of Jilin University, Changchun 130000, China

[4] Department of Computer Science, Carnegie Mellon University, PA 15213, USA

[5] Department of Statistics, Harvard University, Cambridge, MA 02138 USA

[6] MidSouth Bioinformatics Center and Joint Bioinformatics Ph.D. Program of University of Arkansas at Little Rock and Univ. of Arkansas Medical Sciences. 2801 S. Univ. Ave., Little Rock, 72204, USA

[7] University of Arkansas at Little Rock, Little Rock, AR 72204, USA

[*] Corresponding author, E-mail: guanrenchu@jlu.edu.cn

The supplementary information includes:

Additional file 1. Figure S1 The word cloud results to analyze relationships between diabetes, obesity and other diseases from 2007 to 2016

Additional file 1. Table S2 Clinical Report Proofs on the Discoveries about Diabetes and Other Diseases

Additional file 1. Table S3 Clinical Report Proofs on the Discoveries about Obesity and Other Diseases

Additional file 1. Figure S4 The Research Hotspots of ten years (2007 ~ 2016)

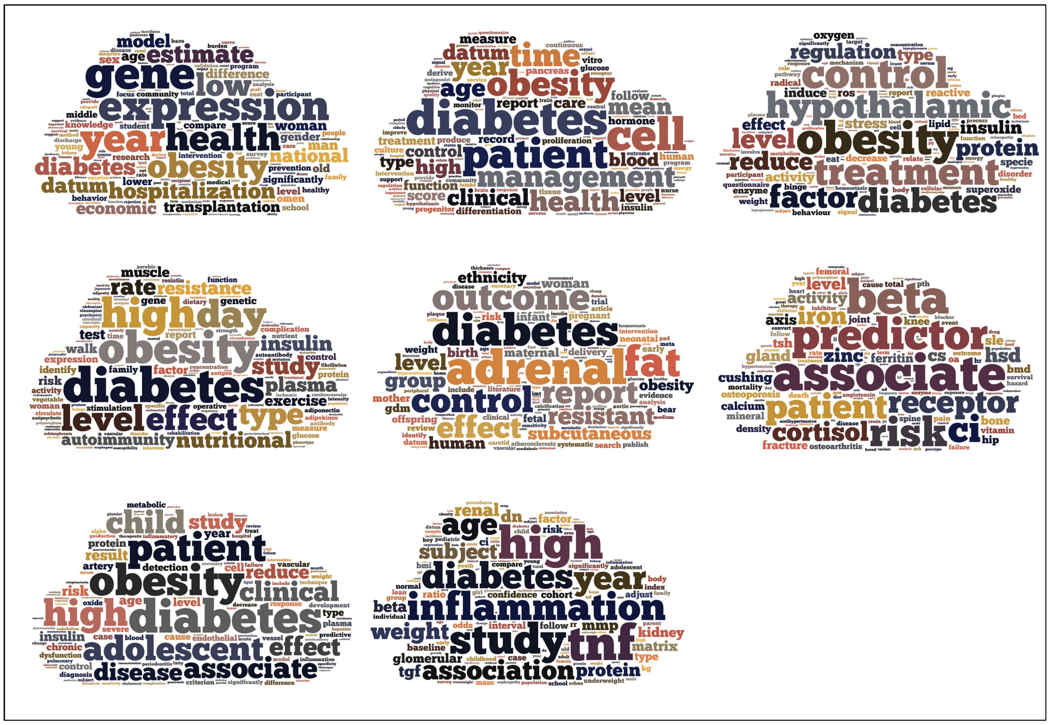

Additional file 1. Figure.S1.a The word cloud results of 2007

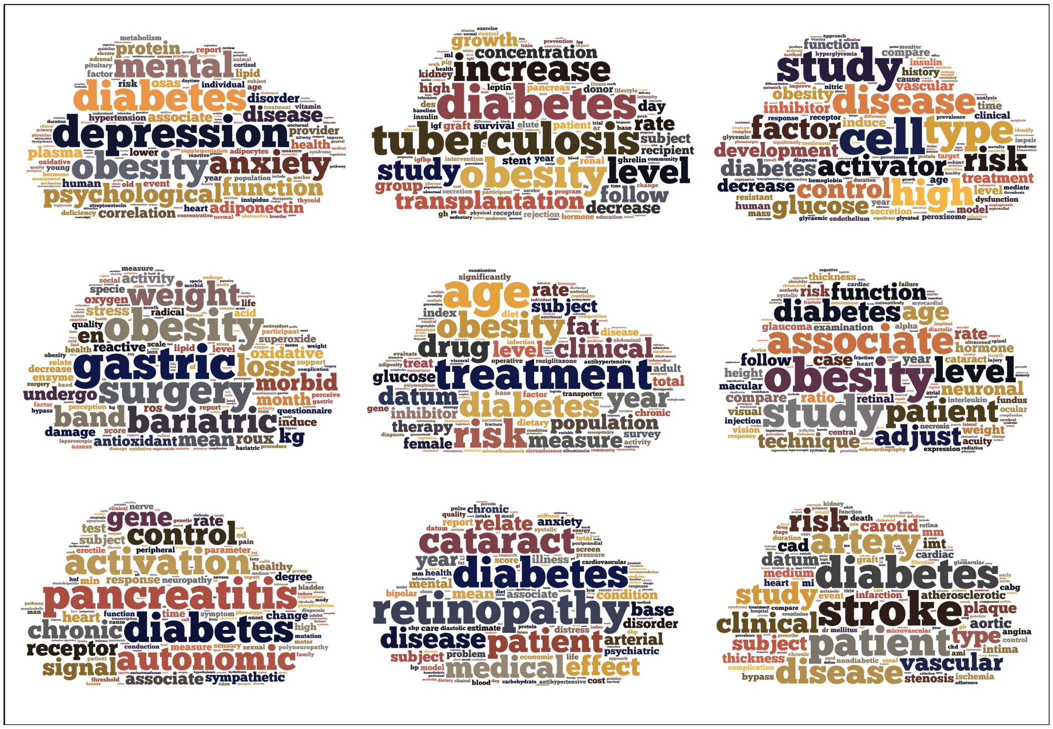

Additional file 1. Figure.S1.b The word cloud results of 2008

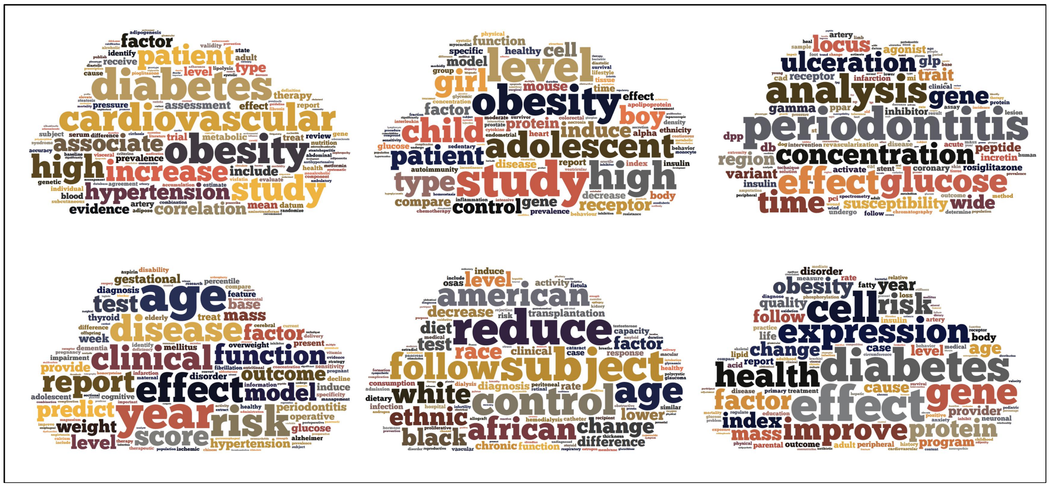

Additional file 1. Figure.S1.c The word cloud results of 2009

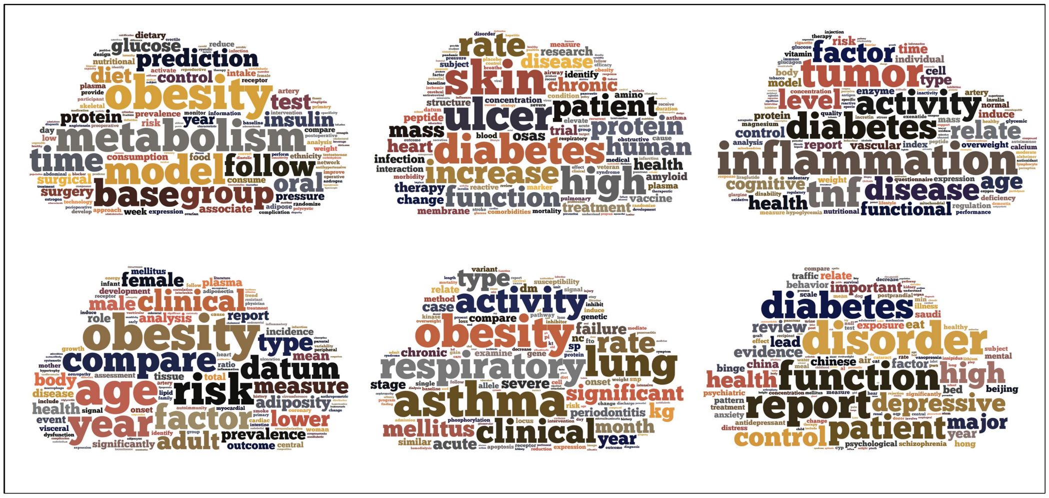

Additional file 1. Figure.S1.d The word cloud results of 2010

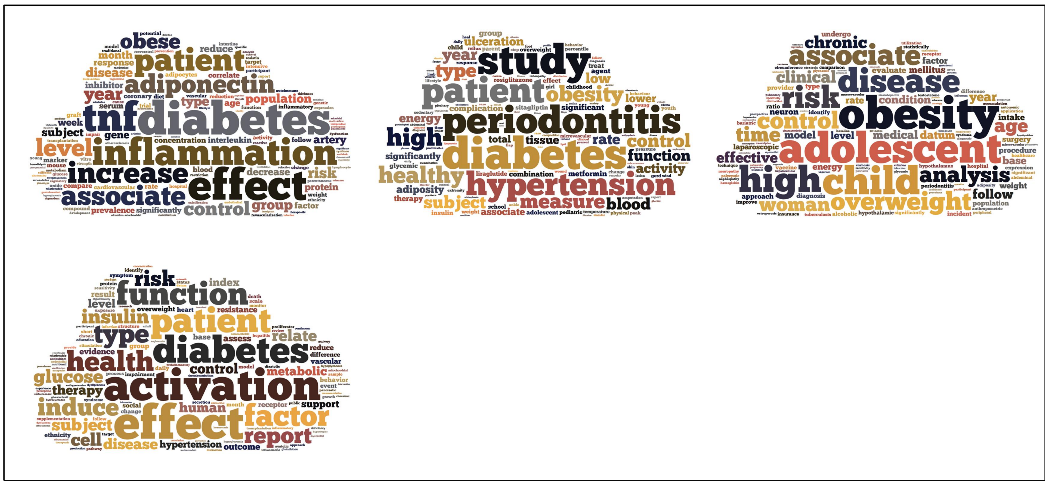

Additional file 1. Figure.S1.e The word cloud results of 2011

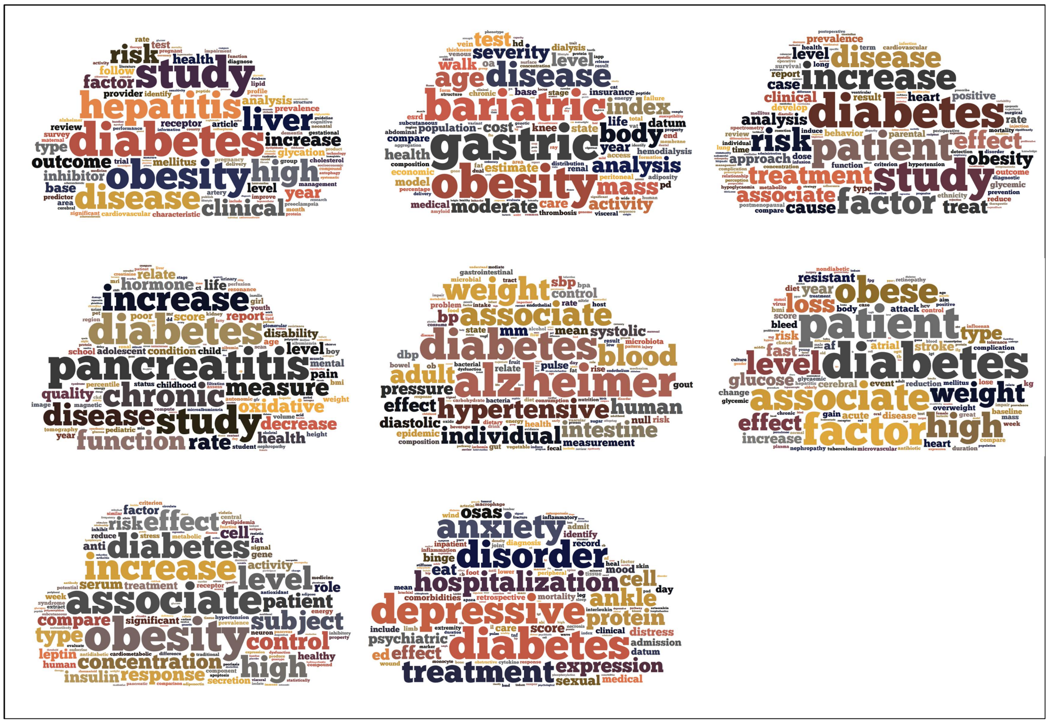

Additional file 1. Figure.S1.f The word cloud results of 2012

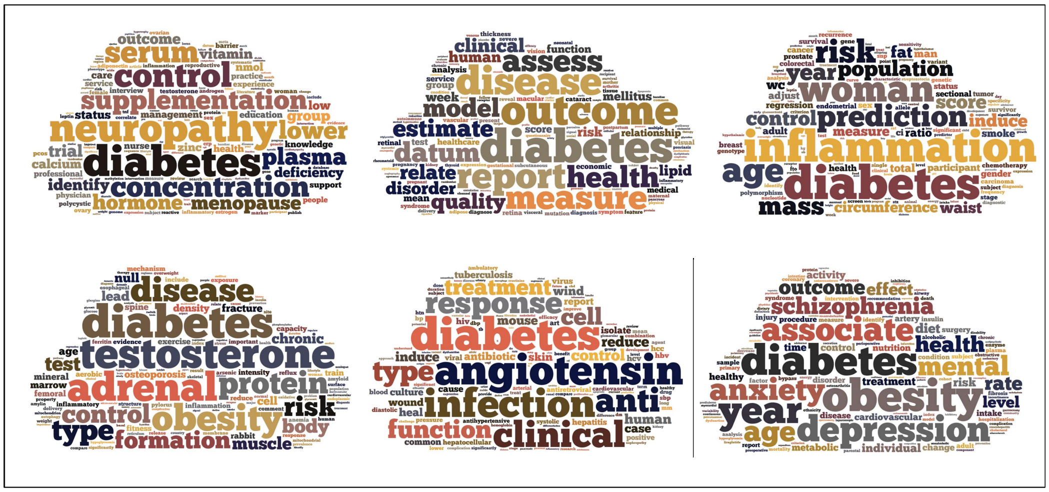

Additional file 1. Figure.S1.g The word cloud results of 2013

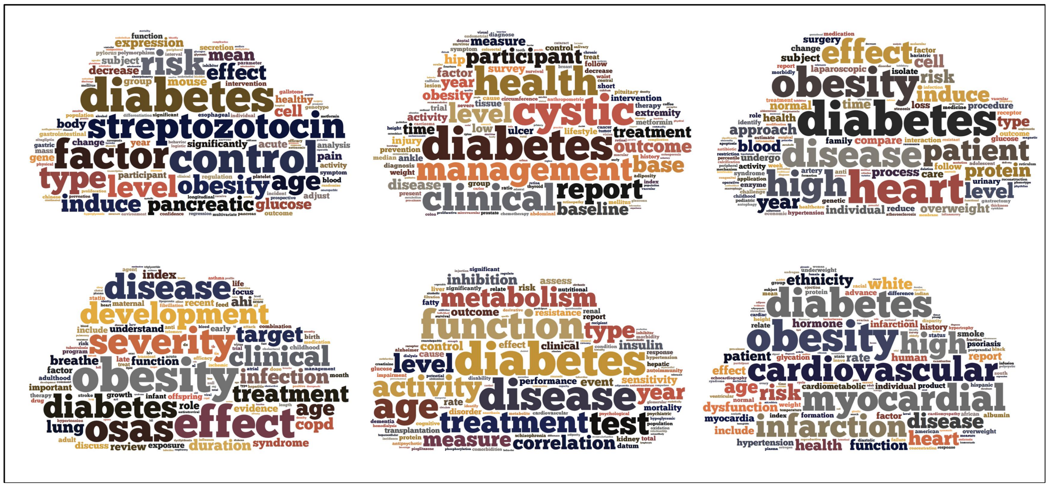

Additional file 1. Figure.S1.h The word cloud results of 2014

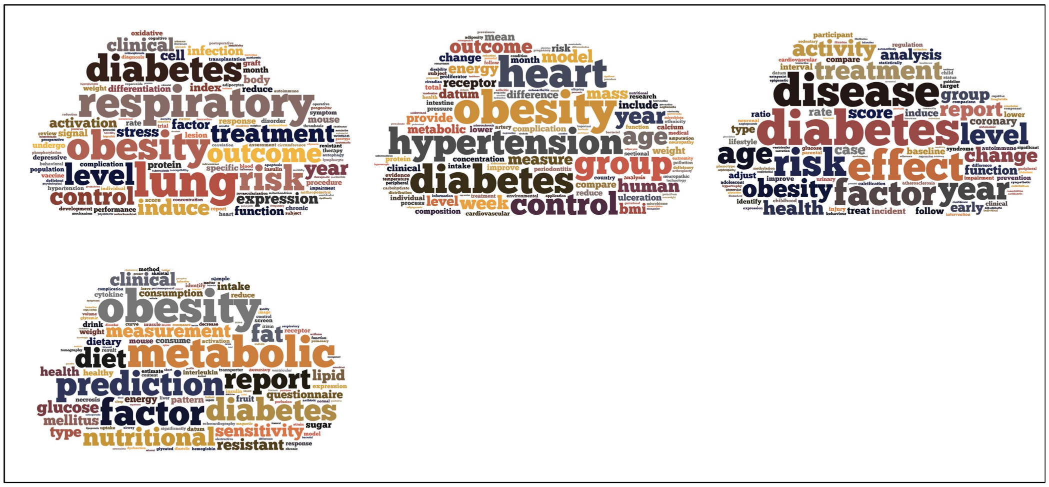

Additional file 1. Figure.S1.i The word cloud results of 2015

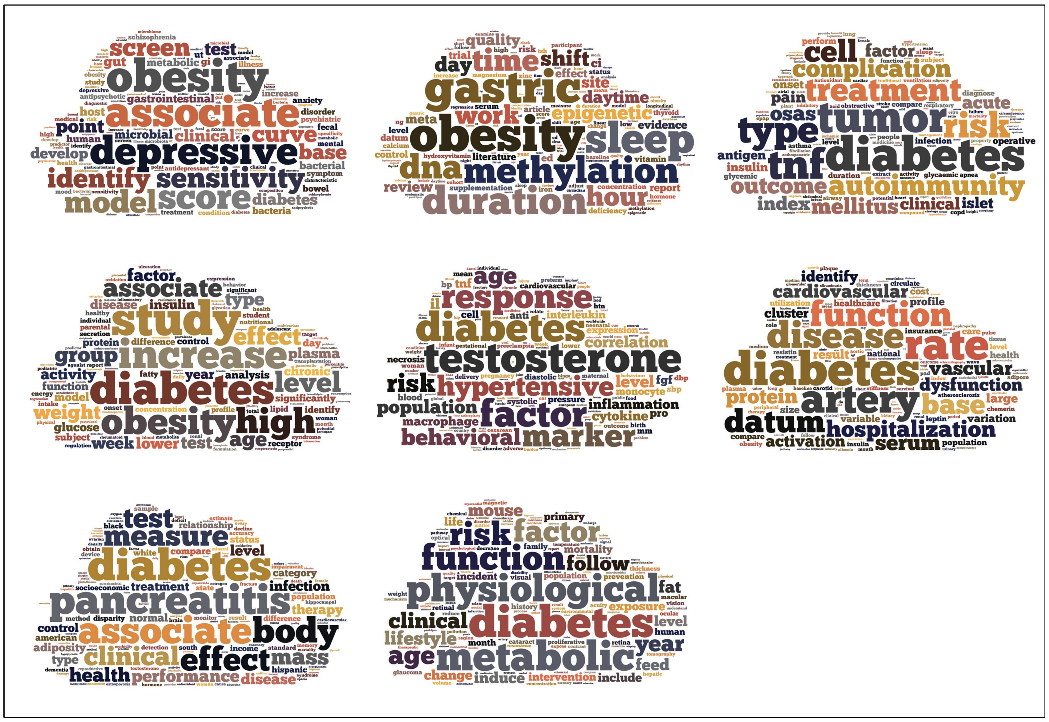

Additional file 1. Figure.S1.j The word cloud results of 2016

Additional file 1. Figure S1 The word cloud results for analyzing relationships between diabetes, obesity and other diseases from 2007 to 2016

Additional file 1. Table S2 Clinical Report Proofs on the Discoveries about Diabetes and Other Diseases

Diseases	Quotes	Clinical Report
anxiety	It is important to consider the impact of diabetes on quality of life as well as the development of mental health problems related to diabetes distress, fear of hypoglycemia (and hyperglycemia), symptoms of **anxiety**, disordered eating behaviors as well as eating disorders, and symptoms of depression.	Standards of Medical Care in Diabetes - 2016 [1]
cataract	Glaucoma, **cataract**, and other disorders of the eye occur earlier and more frequently in people with diabetes.	
cystic disease	Cystic fibrosis–related diabetes (CFRD) is the most common comorbidity in people with **cystic fibrosis**, occurring in about 20% of adolescents and 40–50% of adults.	
depression	**Depression** affects 20–25% of people with diabetes.	
myocardial infarction	Individuals with both diabetes and major depressive disorder have a twofold increased risk for new onset **myocardial infarction** compared with either disease state alone.	
retinopathy	Diabetic **retinopathy** is a highly specific vascular complication of both type 1 and type 2 diabetes.	
stroke	Older individuals with diabetes have higher rates of premature death,	

hypertension	functional disability, and coexisting illnesses, such as **hypertension**, coronary heart disease, and **stroke**, than those without diabetes.	
hepatitis	Compared with the general population, people with type 1 or type 2 diabetes have higher rates of **hepatitis** B.	
inflammation	Genetically determined β-cell function and insulin resistance associated with infection and **inflammation** may also contribute to the development of CFRD.	
heart disease	Almost 50% of patients with type 2 diabetes will develop **heart failure**.	
liver disease	In a prospective analysis, diabetes was significantly associated with incident nonalcoholic chronic **liver disease** and with hepatocellular carcinoma.	
lung disease	Diabetes in this population (CFRD), compared with individuals with type 1 or type 2 diabetes, is associated with worse nutritional status, more severe inflammatory **lung disease**, and greater mortality.	
schizophrenia	Severe mental disorder that includes **schizophrenia**, bipolar disorder, and depression is increased 1.7-fold in people with diabetes.	
skin ulcer	Foot **ulcers** and amputation, which are consequences of diabetic **neuropathy** and/or peripheral arterial disease (PAD), are common and represent major causes of morbidity and mortality in people with diabetes.	
neuropathy		
adrenal disease	Adults who develop type 1 diabetes may develop additional autoimmune disorders including thyroid or **adrenal dysfunction** and celiac disease.	
cardiovascular disease	Because patients with diabetes have greatly increased risk for **cardiovascular disease**.	
Alzheimer's disease	The reverse is also true: people with Alzheimer dementia are more likely to develop diabetes than people without **Alzheimer** dementia.	
pancreatitis	Diabetes mellitus (DM) is a common complication of chronic **pancreatitis** (CP) and increases the mortality.	Risk Factors for Diabetes Mellitus in Chronic Pancreatitis: A Cohort of 2011 Patients [2]
periodontitis	**Periodontitis** is more common in diabetics, and occurs with increased severity when the diabetes is uncontrolled.	Diabetes and Periodontitis: A medical perspective [3]
respiratory disease	Diabetes in this population is associated with worse nutritional status, more severe inflammatory lung disease, and greater mortality from **respiratory failure**.	Standards of Medical Care in Diabetes – 2014 [4]

tuberculosis	Diabetes is a known risk factor for **tuberculosis** and is associated with poorer **tuberculosis** outcomes, while tuberculosis is associated with worsening glycaemic control.	Global Report on Diabetes [5]
tumor	Laboratory and clinical evidence suggest that diabetes caused by pancreatic cancer is due to cytokines produced by the **tumor** rather than secondary to endocrine pancreatic tissue invasion and damage.	Diabetes and cancer [6]
OSAS	**OSAS** is a common disorder characterized by repetitive episodes of partial or complete obstruction of the upper airway during sleep and increased respiratory effort. This syndrome can lead to the development of obesity and diabetes.	Obesity, diabetes and OSAS induce of sleep disorders: Exercise as therapy [7]
hypothalamic disease	**Hypothalamic inflammation** links central insulin resistance to diabetes.	One Step from Prediabetes to Diabetes: Hypothalamic Inflammation? [8]

Additional file 1. Table S3 Clinical Report Proofs on the Discoveries about Obesity and Other Diseases

Diseases	Quotes	Clinical Report
depression	Obese adults are more likely to have **depression**, **anxiety** and other mental health.	The State of Obesity 2016 [9]
anxiety		
asthma	Being overweight or obese can put children at a higher risk for health problems such as **heart disease**, **hypertension**, type 2 diabetes, stroke, cancer, **asthma** and osteoarthritis — during childhood and as they age.	
heart disease		
hypertension		
liver disease	Up to 25 percent of adults have nonalcoholic fatty **liver disease** (NFLD), which can lead to liver damage (cirrhosis) or the need for transplants.	
hepatitis	In the present study, our results further suggested that obesity was significantly associated with non-response to **hepatitis** B vaccination.	Hepatitis B vaccine response in obesity: A meta-analysis [10]
gastric disease	It is well recognized that Helicobacter pylori infection, dietary habits, smoking, and obesity are risk factors for the development of **gastric cancer**.	A case report of chylous ascites after gastric bypass for morbid obesity [11]
lung disease	Forno et al showed that obese children had evidence of dysanapsis (a dissociation of lung airway growth with lung size) that may be contributing to **lung disease** in obese children.	Beyond BMI: Obesity and Lung Disease [12]
myocardial infarction	Epidemiological evidence suggests that overweight and obesity have been associated with acute **myocardial infarction** (AMI).	The incidence of acute myocardial infarction in relation to overweight and obesity: a meta-analysis [13]

respiratory disease	Obesity has a direct causal effect on some **respiratory diseases**, namely OSA and OHS.	Obesity, respiratory disease and pulmonary infections [14]
tuberculosis	Mounting data have revealed that body mass index (BMI) is inversely associated with risk of active **tuberculosis**.	Association of Obesity, Diabetes, and Risk of Tuberculosis: Two Population-Based Cohorts [15]
hypothalamic disease	Collectively, this work identifies a potential link between obesity and **hypothalamic injury** in humans as well as animal models.	Obesity is associated with hypothalamic injury in rodents and humans [16]
cardiovascular disease	Obesity increases the risk of **cardiovascular disease** and premature death.	Mechanisms linking obesity with cardiovascular disease [17]
OSAS	One of the conditions whose prevalence is increased by obesity in childhood is the **obstructive sleep apnea syndrome** (**OSAS**).	Obesity and obstructive sleep apnea syndrome in children: A tale of inflammatory cascades [18]
adrenal disease	There are few studies reporting on higher rates of overweight and obesity among children with **CAH** (**Congenital adrenal hyperplasia**).	Obesity Among Children and Adolescents With Classic Congenital Adrenal Hyperplasia Due to 21-Hydroxylase Deficiency [19]
schizophrenia	With increased rates of obesity in **schizophrenia**, it is important to highlight the potentially deleterious effect of obesity on cognition.	Unraveling the relationship between obesity, schizophrenia and cognition [20]

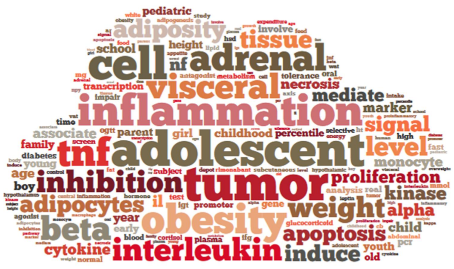

a. Research Hotspots of 2007

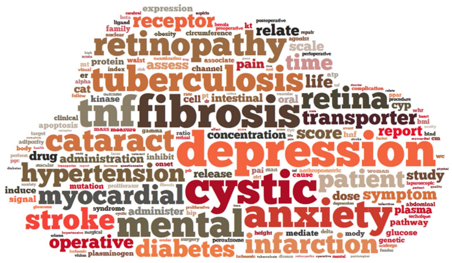

b. Research Hotspots of 2008

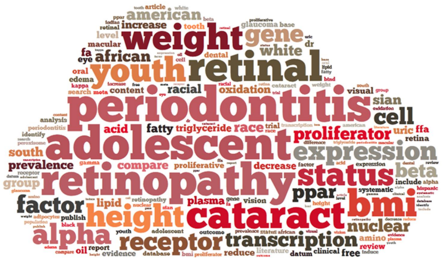

c. Research Hotspots of 2009

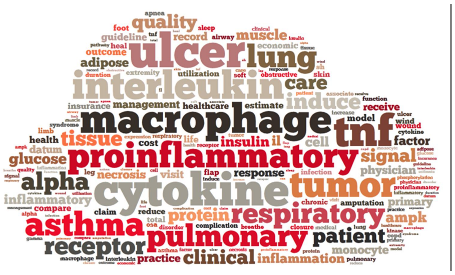

d. Research Hotspots of 2010

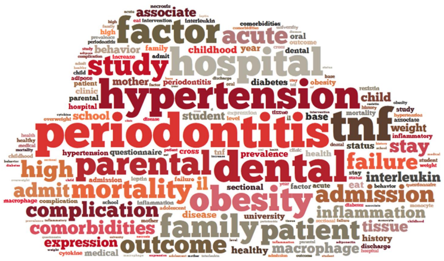

e. Research Hotspots of 2011

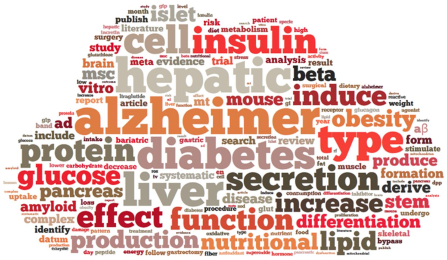

f. Research Hotspots of 2012

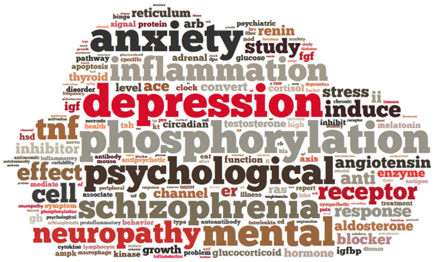

g. Research Hotspots of 2013

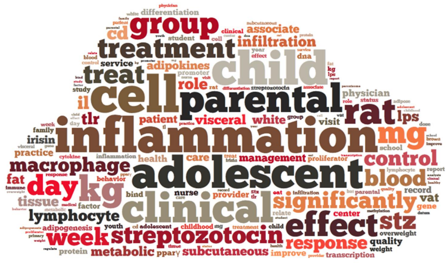

h. Research Hotspots of 2014

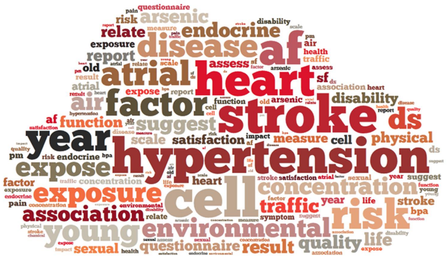

i. Research Hotspots of 2015

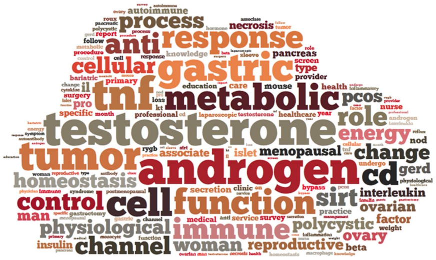

j. Research Hotspots of 2016

Additional file 1. **Figure S4 The Research Hotspots of the last ten years (2007 ~ 2016)**

References

[1] American Diabetes Association. Standards of medical care in diabetes-2016. Diabetes care, 2016; 39(1): S1-S112.

[2] Pan J, Xin L, Wang D, et al. Risk factors for diabetes mellitus in chronic pancreatitis: a cohort of 2011 patients [J]. Medicine, 2016, 95(14).

[3] Sharma M, Jindal R, Siddiqui M A, et al. Diabetes and Periodontitis: A medical perspective [J]. Journal of the International Clinical Dental Research Organization, 2016, 8(1): 3.

[4] American Diabetes Association. Standards of medical care in diabetes—2014 [J]. Diabetes care, 2014, 37(Supplement 1): S14-S80.

[5] World Health Organization. Global report on diabetes [M]. World Health Organization, 2016.

[6] Vigneri P, Frasca F, Sciacca L, et al. Diabetes and cancer [J]. Endocrine-related cancer, 2009, 16(4): 1103-1123.

[7]Alves E S, Lira F S, Santos R V T, et al. Obesity, diabetes and OSAS induce of sleep disorders: exercise as therapy [J]. Lipids in health and disease, 2011, 10(1): 148.

[8]Cai D. One step from prediabetes to diabetes: hypothalamic inflammation? [J]. 2012.

[9] Trust for America's Health. The State of Obesity: 2016. Robert Wood Johnson Foundation, 2016; September: 1-143.

[10] Fan W, Chen X, Shen C, et al. Hepatitis B vaccine response in obesity: A meta-analysis [J]. Vaccine, 2016, 34(40): 4835-4841.

[11] Capristo E, Spuntarelli V, Treglia G, et al. A case report of chylous ascites after gastric bypass for morbid obesity [J]. International journal of surgery case reports, 2016, 29: 133-136.

[12] Peters U, Suratt B T, Bates J H T, et al. Beyond BMI: Obesity and Lung Disease [J]. Chest, 2017.

[13] Zhu J, Su X, Li G, et al. The incidence of acute myocardial infarction in relation to overweight and obesity: a meta-analysis [J]. Archives of medical science: AMS, 2014, 10(5): 855.

[14] Fernandez C, Manuel A. Obesity, respiratory disease and pulmonary infections [J]. Annals of Research Hospitals, 2017, 1(5).

[15] Lin H H, Wu C Y, Wang C H, et al. Association of obesity, diabetes, and risk of tuberculosis: two population-based cohorts [J]. Clinical Infectious Diseases, 2017.

[16]Thaler J P, Yi C X, Schur E A, et al. Obesity is associated with hypothalamic injury in rodents and humans [J]. The Journal of clinical investigation, 2012, 122(1): 153-162.

[17]Van Gaal L F, Mertens I L, Christophe E. Mechanisms linking obesity with cardiovascular disease [J]. Nature, 2006, 444(7121): 875.

[18]Bhattacharjee R, Kim J, Kheirandish‐Gozal L, et al. Obesity and obstructive sleep apnea syndrome in children: a tale of inflammatory cascades [J]. Pediatric pulmonology, 2011, 46(4): 313-323.

[19]Völkl T M K, Simm D, Beier C, et al. Obesity among children and adolescents with classic congenital adrenal hyperplasia due to 21-hydroxylase deficiency [J]. Pediatrics, 2006, 117(1): e98-e105.

[20]Rashid N A A, Lim J, Lam M, et al. Unraveling the relationship between obesity, schizophrenia and cognition [J]. Schizophrenia research, 2013, 151(1): 107-112.
